# Revealing Nuanced Relationships Among Cognitive Test Anxiety, Motivation, and Self-Regulation Through Curvilinear Analyses

**DOI:** 10.3389/fpsyg.2020.01141

**Published:** 2020-06-15

**Authors:** Jerrell C. Cassady, W. Holmes Finch

**Affiliations:** Department of Educational Psychology, Ball State University, Muncie, IN, United States

**Keywords:** cognitive test anxiety, self-regulated learning, motivation, goals, expectancy

## Abstract

Student motivational profiles and self-regulated learning strategies are significant influences on overall academic success in university settings. Test anxiety has been repeatedly linked to maladaptive learning strategies and ineffective motivational frameworks. However, the results in the field have been inconsistent with respect to the precise interactions among these variables. This study employed anonymous responses from a group of volunteer students in a mid-sized Midwestern United States university, serving a primarily Caucasian and female population with an average age of 20 years. Adopting a curvilinear analytic design, this study attempted to examine the relationships among these common domains of inquiry into student thriving. The results of this study provide insights examining under which conditions cognitive test anxiety is most likely to be heightened or diminished. The results demonstrated that levels of test anxiety were greatest when (a) learners adopted primarily extrinsic or primarily intrinsic goal orientations, (b) academic tasks where the outcome was uncertain, (c) learners adopted passive learning strategies, and (d) learning strategies were more personally involved (as opposed to externalized study behaviors). Our results add to the field by identifying curvilinear models provide greater utility in identifying the relationships among these critical emotional and cognitive factors in academic settings. Furthermore, we advocate for employing identification and intervention strategies that recognize individually specific profiles of interactions among test anxiety, motivation, and self-regulation to promote more optimal success in supporting learners in university settings.

## Introduction

In contemporary educational settings, the success and thriving of students is of critical concern to university leaders as rates of retention and graduation are challenged across all sectors (Cheslock and Kroc, 2012). With more students gaining access to post-secondary education, the initial readiness to perform in university curricula has been demonstrated to be more varied than in any prior generation. To that effect, support mechanisms have been increasingly proposed to support those students in meeting the challenges they face in academic, emotional, and financial domains (Heller and Cassady, 2017). While we recognize all these domains promote challenges to optimal performance, our attention centers on the impact of negative affective experiences triggered by stressors in academic settings (e.g., Pekrun, 2006; Putwain, 2007). Research in the field illustrates between 20 and 45% of students experience debilitating emotional states (e.g., anxiety, depression) that impact their performance in universities (Kessler et al., 2005). A related finding in this domain is that these indicators of academic anxieties in college campuses are good indicators of students who currently experience or are at heightened risk to develop anxiety and/or depression (Cassady et al., 2019).

These growing trends in the field have largely driven our work, which involves three primary goals in this domain: (a) effectively identify students at-risk for maladaptive affective experiences such as test anxiety, (b) isolate contributing factors that promote deleterious performance due to test anxiety, and (c) promote intervention efforts that can support thriving. Over the course of 25 years of research in this domain, attention to cognitive test anxiety and academic behaviors has repeatedly demonstrated that elevated levels of test anxiety are clearly related to key issues in university success such as motivation and study skills. However, recent trends in the field pointing to moderating variables have sparked renewed interest in reviewing the hypothesis that test anxiety may have some facilitative or functional aspects. To that end, our attention has turned to examining more nuanced relationships among motivation, self-regulation, and test anxiety.

### Test Anxiety

Test anxiety influences learners in academic settings through the beliefs, behaviors, and eventual performance differences noted for people with varied levels of experienced affective response in evaluative settings (Cassady, 2010). Research has demonstrated that learners adopt failure accepting and task avoidance motivational sets (Zeidner and Matthews, 2005), are prone to cognitive distractions during both test preparation and test performance phases, and have difficulty with self-regulatory skills such as organization, time management, attentional control, and effective study strategies (Bar-Tal et al., 1999; Eysenck et al., 2007; Cassady, 2010). There are at least two dimensions of test anxiety: emotionality (or physiological) and cognitive test anxiety (or worry; Liebert and Morris, 1967), with more recent models attempting to validate a third aspect (social; Lowe et al., 2008). However, the findings focused on social test anxiety have not effectively determined if social aspects of test anxiety are a clearly distinct factor, or a significantly important environmental influence that promotes test anxiety in the other two domains.

The emotionality component of test anxiety is generally context-specific and is commonly identified through heightened physiological arousal (e.g., heart rate, nausea, agitation; Cassady, 2010). The worry component, or cognitive test anxiety, is characterized by poor processing efficiency (i.e., cognitive overload), unproductive cognitive distractions, and perseveration on fear of failure (Rojas and Furlan, 2017). While most models of test anxiety identify that cognitive test anxiety and emotionality are related, interact with one another, and may best be explained through an additive relationship (Zohar, 1998), the data generally support the conclusion that cognitive test anxiety is more directly related to performance decrements (Hembree, 1988; Cassady, 2004) and stable over time (Cassady, 2001).

Several accounts for the mechanism through which cognitive test anxiety drives down performance have been identified, pointing to reduced working memory capacity (Mowbray, 2012), inability to organize cognitive information effectively (Naveh-Benjamin et al., 1987; Bar-Tal et al., 1999), and perseveration on negative self-thoughts or engaging in avoidance strategies that limit cognitive resources available to engage in the task at hand (Sarason, 1986; Raffety et al., 1997; O’Carroll and Fisher, 2013). The Attentional Control Theory (ACT; Eysenck et al., 2007) provides an effective model that provides a comprehensive explanatory framework for these findings to date. One of the key premises offered in ACT is that when test anxiety is heightened, learners overload their cognitive resources through an inability to inhibit distracting thoughts (e.g., worry over fear of failure). This leads to cognitive overload due to the increase in extraneous processing (e.g., Mayer, 2014), limiting the ability to engage in goal-directed cognition (e.g., effective study strategies, focus during tests). Another strong prediction offered by ACT is that the negative impact on learner performance is primarily an operation of reduced processing efficiency, not processing efficacy (Eysenck and Calvo, 1992). That is, ACT does not presume that individuals with test anxiety are necessarily incapable of activating effective coping strategies or cognitive operations. Wong et al. (2013) provided compelling support for this efficiency proposal, illustrating that as basic cognitive tasks became more challenging, significant differences in time taken to respond (but not actual performance) were noted between learners with high and low levels of trait anxiety.

The standard approach to analyzing outcomes in test anxiety research has relied heavily on basic linear models of analyses. However, one of the earliest (and most durable) conceptions for stress and anxiety is the Yerkes-Dodson Law (Yerkes and Dodson, 1908), which proposed that there is an optimal point of arousal (or stress) at which performance is maximized – but once that threshold is crossed, performance outcomes decline rapidly. One justification for the focus on linear effects is that “anxiety” is a maladaptive level of “stress,” so the Yerkes-Dodson threshold has already been crossed and any degree of anxiety is maladaptive. However, this overlooks the broad tendency in the field to explain differential influences for students with “levels” of anxiety (e.g., Thomas et al., 2017a). Moreover, the results have been clear that there are interactions in student outcomes that would be missed without attention to interactions or non-linear trends. For instance, Mattarella-Micke et al. (2011) demonstrated that the influence of cortisol (a measure generally indicating high stress) on performance was only understood when examining the interactive relationships among working memory and math anxiety in those tasks. Similarly, Stowell et al. (2008) found that explaining the influence of cortisol, test anxiety, and coping strategies on course performance and affective states was only possible when examining moderation effects of the coping mechanisms in the specific context. These trends are even more variable in contexts that openly identify aspects of “facilitative anxiety,” which are levels or behaviors connected to anxiety over examinations that do not drive a negative affective experience (Raffety et al., 1997). Taken together, the research suggest that much of the research in the field may have missed the nuance of the relationships among test anxiety, motivational variables, student behaviors, and performance.

### Self-Regulated Learning

Self-regulated learning strategies are a collection of cognitive and behavioral strategies that promote optimal success by ensuring that learning activities are strategic and targeted planning, self-monitoring, and effortful control over cognitive processes in directing efforts toward attaining achievement goals (Zimmerman, 2002; Schunk and Zimmerman, 2003). The value of SRL has become increasingly important in educational contexts as students have been progressively expected to engage in more self-directed learning experiences in educational settings – that is, as instruction becomes less teacher-centered (Bjork et al., 2013). To illustrate the power of SRL in contemporary settings, Duckworth and Seligman (2005) demonstrate that self-discipline (i.e., monitoring and maintaining effort control) was twice as effective in predicting academic performance than IQ. Kitsantas and Zimmerman (2009) also showed that SRL mediated the positive relationship between homework activity and achievement, demonstrating that actively monitoring and controlling the independent learning experiences was necessary to explain the performance gains. While the evidence is strong that effective use of self-regulated learning strategies and effective learning techniques can promote positive outcomes, it has also become evident that students often fail to engage in quality strategies due to their perceived utility, lack of skill practice, or simple preference for less effective methods (e.g., Dunlosky et al., 2013). While the bulk of attention in the SRL domain has targeted cognitive and metacognitive control, Pintrich (2004) argued effectively that effective models need to also take into consideration learners’ regulation of affect, behavior, and the context within which they are operating.

Following Pintrich (2004) call for attention to the broader regulatory functions involved in self-regulation, the Emotional Information Processing model (Cassady and Boseck, 2008; Cassady and Thomas, 2020) proposed an iterative process for (a) encoding and interpreting internal and external cues in academic settings, (b) developing and evaluating goals to respond to the perceived context, and (c) implementing solutions to achieve established goals. Central to this model is the recognition that the learner’s representation of stressors in academic settings dictates the goals established, appraisal of the likelihood of managing those stressors, and the coping mechanisms that will be adopted to manage the context in an effort to reduce the level of perceived threat. Similarly, Gross (1998, 2015) Process Model for emotion regulation identified there are two broad approaches learners employ to manage perceived stress or threat in academic settings: (a) activate proactive or problem-focused coping strategies that aim to improve success directly, or (b) change the emotional set or mitigate the severity of the emotional response through emotion regulation strategies.

The operationalization of effective SRL in response to stressors can generally be identified by examining the coping strategies learners employ when navigating the stressful or threatening academic context. Lazarus and Folkman (1987) foundational Transactional Stress and Coping model and more recent conceptualizations such as the Self-Referent Executive Processing model (Wells and Matthews, 1996; Putwain, 2019) demonstrated that the individual’s appraisal of perceived stressors or threats as well as their metacognitive knowledge regarding potential coping strategies determine the probable strategies that would be employed. The research that has followed in this line has classified three broad forms of coping. Problem-focused, or adaptive, coping are those strategies that are focused on promoting positive study behaviors or actively pursuing strategies that will promote competency in the domain where perceived ability is in question (e.g., Zeidner and Saklofske, 1996). Examinations of the interactions among active coping strategies and academic buoyancy demonstrate that the maladaptive outcomes associated with high test anxiety are significantly reduced when students engage in effective test preparation coping strategies and maintain a higher degree of buoyancy (Putwain et al., 2016). Avoidance strategies are coping mechanisms such as procrastination, withdrawal, or self-handicapping that remove the learner from the context that promotes the stress (Kalechstein et al., 1989; Cassady and Thomas, 2020). Finally, emotion-focused coping strategies target the appraisal of the event itself, attempting to reduce the perceived threat through strategies such as cognitive reappraisal (Brady et al., 2018).

A key debate in the field centers on the utility of the activation of emotion-focused coping strategies. Several studies have demonstrated that learners who rely on strategies that primarily focus on emotion-focused or avoidance coping are maladaptive when compared to problem-focused (or adaptive) coping strategies (Thomas et al., 2017a, b). However, some studies have demonstrated that emotion-focused and avoidance coping strategies can support learners by moderating the influence of cognitive test anxiety (or worry) leading to positive outcomes in affect response (Stowell et al., 2008) and even performance (Brady et al., 2018). However, the positive effects of avoidant or emotional coping strategies are seldom simple effects, revealing their potential is achieved through interactions with other personological and environmental factors (e.g., de la Fuente et al., 2019). Referring back to the EIP framework, our synthesis of the data in the field argues that coping strategies (adaptive, avoidant, emotional) all have potential proactive influence, provided the strategies collectively enable the learner to represent the academic situation as less threatening as well as engage in positive strategies that increase their cognitive and behavioral engagement toward meeting the external needs imposed (Cassady and Thomas, 2020).

### Achievement Motivation

Closely related to the concept of self-regulated learning is the broad domain of achievement motivation. There are many viable models of motivation in the academic context that have been used to explain volitional control effectively, including Weiner (2018) attribution theory, Deci and Ryan (2012) Self-Determination Theory, and Wigfield and Eccles (2002) Expectancy–Value Theory (EVT). Across these models, two primary motivational constructs are central to most treatments of test anxiety, self-regulated learning, and success in university settings. The first motivation theme is the learner’s expectancy for success in the given context. The likelihood that a learner will perform well on a given task, and perform well over a period of time, is greatly enhanced by the belief in their ability to be successful, or self-efficacy (Bandura, 1977; Wigfield and Eccles, 2002). A second general influence on motivation is the underlying impetus that learners focus on when determining perceived value for achieving success in the task. A simplified method for pursuing this second domain includes examining the goal orientations individuals adopt, that is working to achieve success for purposes of satisfying extrinsic or intrinsic interests. It should be stated that although our attention in this study aligns with the EVT model primarily, motivational researchers with preference for SDT models or attribution theories have clearly and effectively explained these constructs from their own perspectives (Wigfield and Eccles, 2002; Weiner, 2018).

#### Expectancy for Success

Bandura (1977) and Bandura and Schunk (1981) work on self-efficacy has been instrumental in demonstrating that learners’ beliefs in their ability to successfully complete a task is instrumental in determining positive academic behaviors and is strongly tied to performance. Simply stated, when students believe they possess the talent and tools necessary to complete a task, they adopt more proactive and adaptive strategies to achieve success. However, when they believe that the task is beyond their skills or abilities, there is a considerable lack of motivational drive to pursue success. Within the framework of this investigation, the data demonstrate that higher levels of self-efficacy are associated with reductions in test anxiety (Brandmo et al., 2019). In cases where the appraisal of either personal attributes or the learning environment suggest to students that success is not likely, motivation to engage in positive academic strategies or SRL wanes (Gorges and Göke, 2015).

Using this framework, researchers have demonstrated strong relationships among expectancy for success and proactive coping, persistence in the face of failure, academic interest, and performance (Bandura and Schunk, 1981; Durik et al., 2015). While the data have been clear in identifying the positive influence of high expectancy ratings (e.g., self-efficacy, confidence, control; Putwain and Aveyard, 2018), Weiner (2018) has also identified that attention to the attributional set of the learner is critical. Specifically, if the expectation for successful performance is based merely on ability, adequate preparation may suffer when compared to attributional styles that presume success is attainable due to diligent effort. In line with this domain of explaining the influence of expectancy for success is the perception of control over the outcomes of an examination, with evidence that higher perceived control led to greater performance particularly when test worry was low to moderate (Putwain and Aveyard, 2018). This line of work has demonstrated further that the impact of perceived control was less instrumental at high levels of worry (Putwain and Aveyard), but additional work has demonstrated that promoting perceptions of control over the situation can also reduce the overall levels of cognitive test anxiety components (Putwain and Prescod, 2018).

##### Goal orientation

There has also been a considerable amount of research examining the influence of achievement goal orientation on learner outcomes (Schunk, 1990; Brandmo et al., 2019). A common theme in research on learners’ motivational approaches to learning from a goal orientation perspective tends to cast dimensional or dichotomous accounts for “types” of goal setting. A dominant orientation toward examining achievement motivation goals focuses on the purpose of the learning experience (from the perspective of the learner). Mastery-focused (or learning) goals have a primary orientation to successfully mastering the content faced by the learner. Conversely, performance goals tend to be focused on the learner demonstrating competence or success in relation to a set standard or in comparison to peers (Shim et al., 2011). Early discussions on mastery vs. performance goal structures often suggested a qualitatively superior status for mastery goals, leading to greater enjoyment, persistence, and eventual long-term success. Research on goal orientations has demonstrated that in general, performance goals are positively related to test anxiety, perhaps exacerbating the situational threat imposed by exam situations (Brandmo et al., 2019). However, continued work in the field has demonstrated that the context matters, demonstrating that performance goals (both approach and avoidance) can be adaptive strategies to promote positive motivational impulses (Brady et al., 2018).

An alternative (but similar) orientation to explaining goal structures focuses on explaining where the primary drive for achieving the goal comes from, and the underlying affective responses to those differentially sourced goals (Pekrun, 2006). Extrinsic goal orientation represents those goals that are established primarily to achieve an externally imposed criterion or satisfaction (e.g., grades, peer approval, access to resources). Conversely, intrinsic goal orientations are those goals that are adopted primarily to satisfy internal needs (e.g., satisfaction, personal growth, skill development; Deci and Ryan, 2012). While educators have been repeatedly coached to prefer intrinsic goal orientations, the research is also mixed in this classification system – essentially generating the conclusion that contextual variations lend themselves to different “optimal” goal orientations, and that the efficacy of those goal orientations are mediated through coping strategies (Brdar et al., 2006).

Our synthesis of the research on these broad motivational constructs highlights once again the importance of person × context interactions (Cassady and Thomas, 2020). Essentially, we argue that reliance on simplified representations of goal orientations (e.g., “intrinsic goals are better than extrinsic”) as well as expectancy frameworks (e.g., “high self-efficacy is always good”) fail to capture the nuances among diverse learners as well as the experiences of individual learners across multiple settings.

## Current Study

While the data in this field has consistently demonstrated connections among these motivational and self-regulatory processes and test anxiety, we have noted a considerable gap in the literature regarding the examination of non-linear relationships. That is, primarily linear relationships have been reported in the literature despite the widely known Yerkes-Dodson law of arousal and performance that is established on findings over 100 years old (Yerkes and Dodson, 1908), which asserted a curvilinear relationship between arousal (stress) and performance. Optimal levels of performance occurred when arousal (stress) was moderate. This explanation is the source of several coping and self-regulation explanations for behavioral exhibition in academic settings related to balancing the level of “pressure” to induce quality performance in academic settings. However, test anxiety research is primarily based on findings centered on linear relationships (Cassady, 2010). Studies that have explored moderation effects among these variables (e.g., Owens et al., 2012) as well as those that identify contexts in which some degree of test anxiety is facilitative (e.g., Raffety et al., 1997; Eysenck et al., 2007) has prompted our attention to examining non-linear relationships among test anxiety, self-regulation, and motivation in a university setting. To the point, our investigation was focused on exploring potential non-linear effects in an attempt to determine if we can identify levels of test anxiety that may serve as activating or facilitative impulses in university settings, and more importantly when that level of test anxiety reaches a tipping point and becomes an entirely negative influence on the learning experience.

## Materials and Methods

This study examined curvilinear relationships among cognitive test anxiety, self-regulation, and motivation (as measured by the Motivated Strategies for Learning Questionnaire, MSLQ) in a university sample (*n* = 298). Participants were enrolled in undergraduate courses in developmental psychology and educational psychology at a mid-sized university in the Midwestern United States. All participants were volunteers, and participation in this survey study was one option of several to satisfy a course requirement. The study protocol and procedures were reviewed and approved as “exempt” by the University Institutional Review Board in accordance with federal guidelines (approval identification number BSU-447466-1). The nature of the data collection process precluded directly identifying gender, race, or age of the students, but prior studies on this population as well as aggregate demographic data on the classes involved in the recruitment indicate that the sample is predominantly Caucasian, female, average age of 20 years, and from the Midwestern United States, consistent with the programs served by the courses. Analyses of the submitted responses demonstrated that fewer than 1% of the observations contained missing values, therefore listwise deletion was employed.

### Measures

#### Motivated Strategies for Learning Questionnaire (MSLQ)

The MSLQ is an 81-item questionnaire addressing student self-reported motivational profiles as well as use of self-regulated learning strategies in academic settings (Pintrich et al., 1991). Students respond to each item after identifying a class or topic of focus – orienting their responses to a specific task for the duration of the scale. Responses are offered on a 7-point Likert-type scale with the extremes marked by “not at all true of me” or “very true of me.”

Fifteen subscales were generated in the original MSLQ and broadly represent motivation and learning strategies. The subscales identified as *Motivation* used in this study include five of the original six offered by Pintrich et al. (1991). In this study, the “Test Anxiety” factor was removed due to the interest in examining test anxiety as a separate variable in the main analyses, leaving (a) internal goal orientation, (b) external goal orientation, (c) task value, (d) control over learning beliefs, and (e) self-efficacy. The nine learning strategies subscales are (a) time and study environment, (b) effort regulation, (c) peer learning, (d) help seeking, (e) rehearsal, (f) elaboration, (g) critical thinking, (h) organization, and (i) metacognitive self-regulation. Pintrich et al. (1991) reported acceptable psychometrics for the 15 scales, with internal consistency estimates across the 15 subscales exceeding a Cronbach’s alpha value of 0.70 on average. Several attempts to generate differential factor structures for the MSLQ subscales (e.g., Cho and Summers, 2012) have revealed no clear agreement in an optimal factor structure that supersedes the initial solution offered in the factor analytic work with the original scale. However, there have been no prior attempts to examine the MSLQ through a multidimensional scaling approach as has been employed in our study.

#### The Cognitive Test Anxiety Scale–Revised (CTAR)

The Cognitive Test Anxiety Scale was originally developed by Cassady and Johnson (2002) as a measure of the cognitive dimension of trait test anxiety, building upon the traditional construct of “worry” in classic representations of test anxiety (Liebert and Morris, 1967; Sarason, 1986). Items in the scale address various aspects of the cognitive test anxiety structure (e.g., worry about doing well on tests, tend to freeze up on tests, forget facts I really know, don’t seem to have much control over my test scores; see Cassady and Finch, 2014, for full scale). Scale validation studies with the original CTAS repeatedly demonstrated problems with the use of “reverse-coded” items in that version (Cassady and Finch, 2014; Thomas et al., 2017a), leading to the creation of a 25-item revised version (CTAR). The CTAR involves no reverse-coded items and was also modified to include items that identify cognitive aspects of test anxiety during all three phases of the learning-testing cycle. Validation studies with the CTAR have demonstrated that the CTAR maintains strong construct validity when compared to other validated measures of test anxiety and related anxiety disorders (Cassady and Finch, 2014, 2015; Cassady et al., 2019), and the current sample demonstrated strong internal consistency for the items once again, *a* = 0.970.

### Analyses

The initial analysis involved the exploration of the dimensional structure of the two domains of the MSLQ (5 motivation subscales; 9 self-regulation subscales) using unfolding multidimensional scaling model (UMSM; Armstrong et al., 2014). UMSMs are a special case of multidimensional scaling, which is a statistical technique designed to reduce dimensionality in a set of variables. Statistical distance values among the variables are calculated, and the resulting weights can then be applied to the variables in order to create scale scores. Variables that are in relatively close in proximity will have similar weights, and those with the largest weights provide primary definition of the resulting scale scores. In the case of the UMSMs used in this study, the R (Version 3.6.0; R Core Team, 2019) smacof library version 2.0 was used to fit the models. The maximum number of iterations was set at 10,000, the lambda (penalty strength) and omega (penalty width) parameters were set to 0.5 and 0.1, respectively, and finally the convergence criterion was 0.000001. Viable two-dimensional representations of both the motivation and self-regulation subscales resulted, yielding four dimensional variables representing students’ responses.

Using these four dimensional variables, we explored linear and curvilinear relationships with test anxiety using Generalized Additive Models (GAM; Hastie and Tibshirani, 1990). GAMs are modeling tools that employ splines to estimate relationships among variables. Splines allow for fitting non-linear complex non-linear relationships between variables. GAMs extend the spline paradigm by identifying the degree of non-linearity that optimizes model fit to the data. For this study, the gam function in the mgcv (version 1.8-31) R library was used for this purpose. A thin plate spline was used in fitting the model, with the generalized cross validation (GCV) score serving to identify the optimal model. The GAMs provided information regarding relationships between each of the dimensional factors and test anxiety. The goal of this analysis was to ascertain the nature of relationships between self-regulation and goal orientation, respectively, with test anxiety, as a way of understanding factors that drive learners’ emotional and cognitive responses to academic challenges or threats. *A priori* hypotheses for the findings of this study included (a) exploration of the structure of the MSLQ would provide a dimensional solution that supported a new and validated approach to represent student motivation and self-regulated learning; and (b) curvilinear modeling would provide superior fit for explaining the relationships among test anxiety and motivational factors (as measured by the MSLQ) as well as self-regulated learning strategies.

## Results

### Unfolding Multidimensional Scaling

**FIGURE 1:**
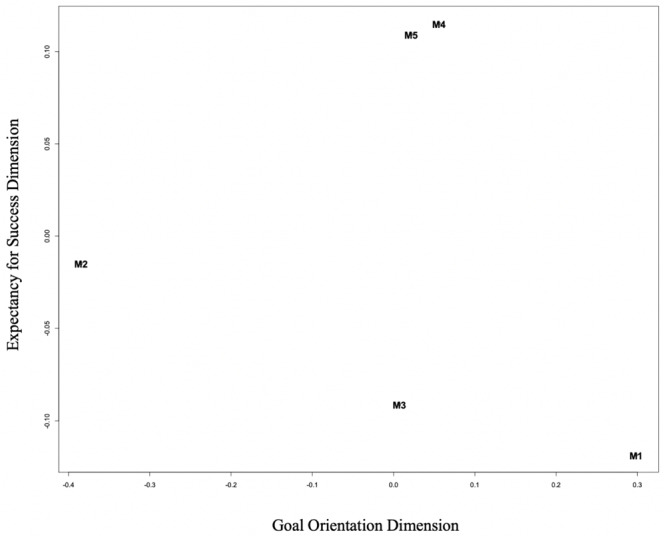
Unfolding model results for MSLQ motivation scales. M1, intrinsic goal orientation; M2, extrinsic goal orientation; M3, task value; M4, self efficacy for learning and performance; M5, control of learning beliefs.

The results of the unfolding model demonstrated that for the motivational variables in the MSLQ (Intrinsic Goal Orientation, Extrinsic Goal Orientation, Task Value, Self-Efficacy for Learning and Performance, Control of Learning Beliefs) two dimensions were detected (Figure 1). The two variables generated that represent the motivational subscales provide dimensional data on “Goal Orientation” and “Expectancy for Success.” Low levels of Goal Orientation (GO) were associated with endorsement of externalized or extrinsic locus of goal construction, whereas high levels demonstrate learners’ affiliation with intrinsic goal orientations. This dimension aligns with a representation of performance (low values) and mastery goal structures. The Expectancy dimensional variable is a simple representation of the learners’ perceived likelihood for success on the target task. High values represent high self-esteem, control over learning outcomes, and confidence in the projected outcome.

**FIGURE 2:**
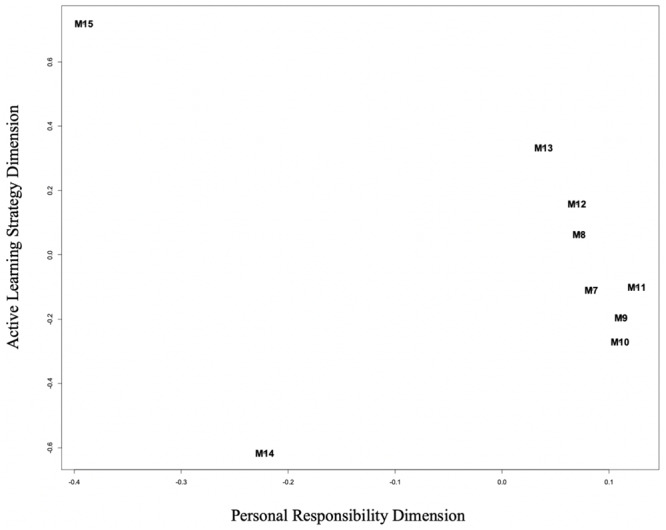
Unfolding model results for MSLQ self-regulated learning scales. M7, rehearsal; M8, elaboration; M9, organization; M10, critical thinking; M11, metacognitive self-regulation; M12, time and study environment; M13, effort regulation; M14, peer learning; M15, help seeking.

With respect to the Self-Regulated Learning components of the MSLQ, an unfolding model was also used, revealing two clear dimensions for those variables (Figure 2), representing “Personal Responsibility for Learning” and “Active Engagement of Learning Strategies.” Personal Responsibility (PR) is focused on the degree to which the self-regulated learning strategy or study skill captured by the MSLQ subscales requires externally supported as opposed to independent self-regulation strategies. This dimension is in line with the SRL vs. ERL framework, with low values on the dimensional variable representing external or social influences in the learning task and high values aligned with strategies and activities that are independent learning approaches. The second self-regulation dimensional variable characterizes the level of Active Learning Engagement (AE) in the learning strategies assessed by the MSLQ. Low values on this dimension represent the more passive learning activities (organizing materials, rote repetition) and high values on the scale are associated with deeply engaged active learning approaches (effort control, elaborative rehearsal).

### Generalized Additive Modeling

Using the four dimensional variables discerned from the unfolding model described above as predictor variables, we explored relationships among cognitive test anxiety and the motivational and self-regulated learning dimensions using Generalized Additive Models (GAMs), with the purpose of ascertaining whether curvilinear functions were present. The resulting GAMs are described below in turn.

#### Cognitive Test Anxiety and Goal Orientation (GO)

**FIGURE 3:**
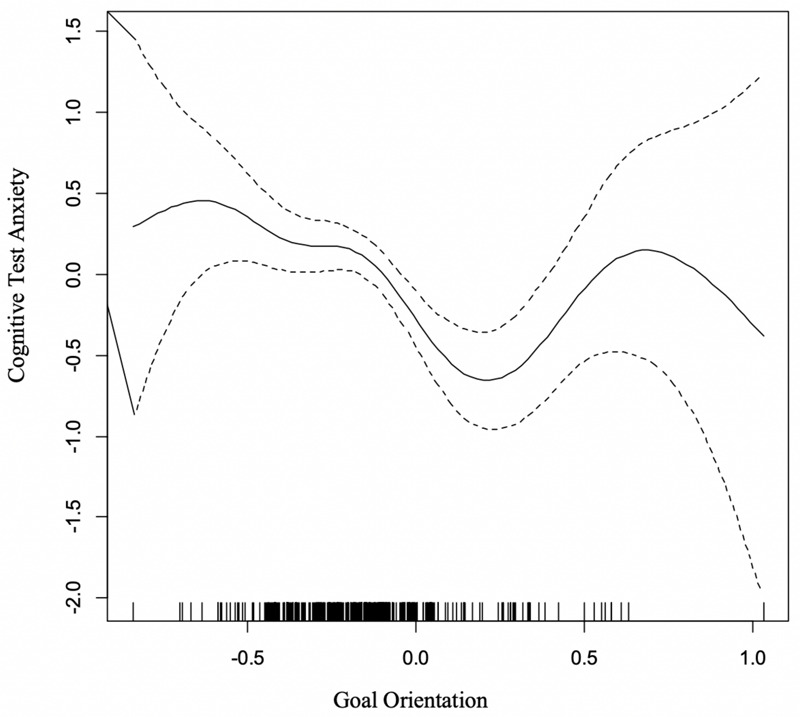
GAM curve relating cognitive test anxiety (*y*-axis) and goal orientation (*x*-axis). High value indicates alignment with Intrinsic Goal Orientation.

The results of the GAM exploring the relationship between CTA and the GO dimensional variable demonstrated a statistically significant curvilinear relationship between GO and CTA [*F*_(__5_._75_, _6_._94__)_ = 4.51, *p* < 0.0001]. The curve in Figure 3 demonstrated that heightened levels of test anxiety (*y*-axis) were present for individuals who reported predominantly extrinsic or intrinsic goal orientations, as illustrated by the heightened anxiety noted on the outer extreme values on the *x*-axis. Reported cognitive test anxiety was lowest for individuals with more even mixtures of goal orientations (mid-level on the *x*-axis). More specifically, the lowest reliably predicted point of anxiety occurs when the mixture between extrinsic and intrinsic goal orientation tendencies slightly favor the intrinsic category. The curvilinear relationship for GO explained approximately 10% of the variance in reported test anxiety.

#### Cognitive Test Anxiety and Expectancy of Success

**FIGURE 4:**
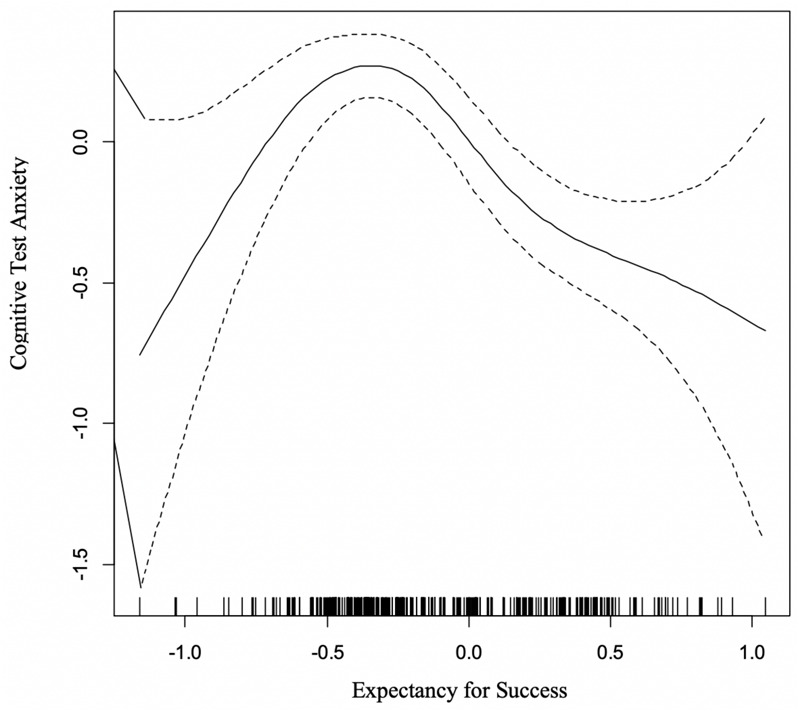
GAM Curve Relating Cognitive Test Anxiety (*y*-axis) and Expectancy for Success (*x*-axis).

The GAM curve for the model relating CTA and the Expectancy dimension appears in Figure 4. This non-linear relationship was statistically significant [*F*_(__3_._63_, _4_._57__)_ = 7.12, *p* < 0.0001], representing an “inverted U” pattern. Test anxiety (*y*-axis) was highest when expected success (*x*-axis) was just below the center point, which is more precisely conveyed as the point of greatest uncertainty. Conversely, lower levels of test anxiety were noted when performance outcomes were at either extreme of the *x*-axis. As such, when success predictions were highly certain (either for success or failure), students reported the lowest degree of cognitive test anxiety. This relationship explained approximately 11% of the variance in test anxiety.

#### Cognitive Test Anxiety and Active Learning Engagement

**FIGURE 5:**
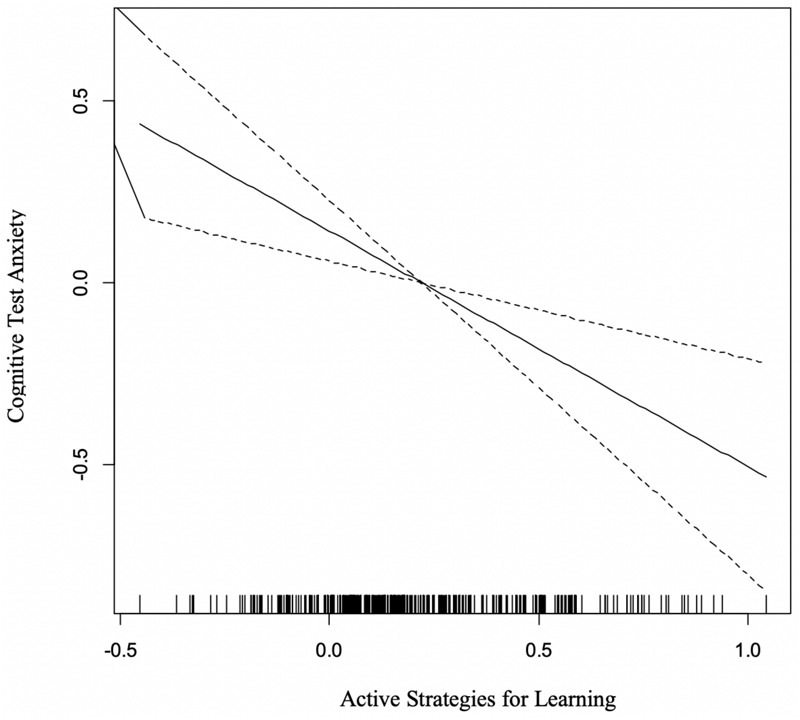
Linear Relationship Cognitive Test Anxiety (*y*-axis) and Active Strategies for Learning (*x*-axis).

There was not a statistically significant curvilinear relationship between cognitive test anxiety and the Active Learning dimension. However, there was a significant inverse linear relationship between these two variables [*F*_(__1_, _1__)_ = 11.62, *p* < 0.0001]. As can be seen in Figure 5, increasing levels of engagement in active learning strategies were directly associated with lower cognitive test anxiety. This relationship accounted for approximately 4% of the variation in cognitive test anxiety scores.

#### Cognitive Test Anxiety and Personal Responsibility for Learning

**FIGURE 6:**
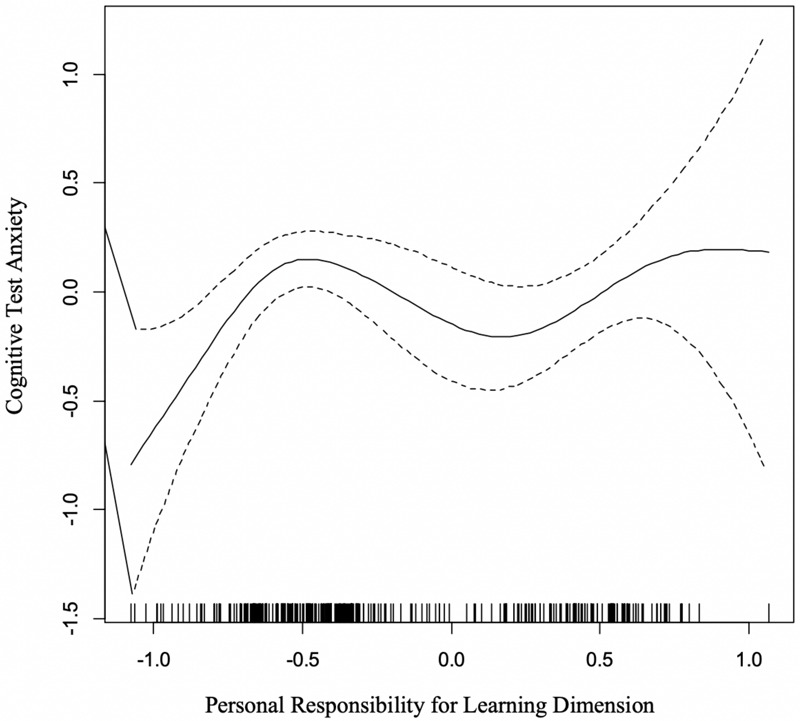
GAM Curve Relating Cognitive Test Anxiety (*y*-axis) and the Personal Responsibility for Learning Activities Dimension (*x*-axis).

The final dimension yielded by the multidimensional scaling, Personal Responsibility for Learning (PRL), had a statistically significant curvilinear relationship with CTA [*F*_(__4_._12_, _5_._14__)_ = 2.46, *p* = 0.03]. Figure 6 shows that lower values on the *x*-axis were associated with lower levels of cognitive test anxiety. In practice, this means that students with a greater reliance or use of socially oriented learning activities tended to have lower levels of CTA. As levels of PRL increased, the level of reported cognitive test anxiety also increased, but overall values of CTA were moderate and stable overall after the first increase noted. This result suggests that above a certain threshold of using socially engaged strategies (e.g., help seeking, peer study activities) there was not a clear direct relationship between PRL and elevated levels test anxiety. However, below that threshold cognitive test anxiety was relatively lower, suggesting that higher reliance on the low-personally responsible activities (cf, socially engaged strategies) or low use of self-regulated strategies high on the PRL dimension are associated with lower test anxiety. This non-linear relationship accounted for approximately 5% of the variation in cognitive test anxiety.

## Discussion

The results of this study provided several insights to the fields of test anxiety and learner outcomes, both in theoretical and practical domains. First, the use of multidimensional scaling to review the data from the MSLQ provides a new approach to represent this commonly used scale in the literature, offering an alternative representation to examine motivational and self-regulatory constructs measured on Pintrich et al. (1991) classic measurement device. From a practical approach to the exploration of test anxiety, the data in this study also confirm our presumption that a high reliance on linear models of examining the relationships among test anxiety and learners’ experiences may have suppressed overall comprehension of the test anxiety construct. Finally, the results of the GAMs elucidate more nuanced relationships among test anxiety and motivation and study behaviors.

### Dimensional Representation for the MSLQ

Over the last 30 years, several attempts to identify a factorial structure that is universally accepted for the MSLQ have been offered. No clear consensus has been achieved in these efforts, leaving most researchers to rely upon ill-fitting solutions or referring to the separate subscales specifically in their investigations. Composite scores for the Motivation domain items separate from the Self-Regulated Learning domain are often used, but typically preclude strong theoretical tests because the subscales that form the composite scores often represent contradictory orientations that do not combine clearly to create an overall “motivation” score (e.g., Extrinsic and Intrinsic Goal Orientations cannot be added to create a conceptual outcome). The dimensional variables that were articulated in the multidimensional scaling analyses provided remarkably clear theoretical consistency with the overall intent of the MSLQ, as reported by Pintrich et al. (1991).

The analysis of the motivational variables used in this analysis revealed a clear two-dimensional representation for the five constructs measured in the MSLQ, providing what we interpreted as a Goal Orientation axis and an Expectancy axis. These two broad constructs are encompassed within all leading theories of motivation, and suggest that the MSLQ motivation scales can inform understanding of motivational goal theories (e.g., Shim et al., 2011; Deci and Ryan, 2012) as well as an expectancy-value orientations (Wigfield and Eccles, 2002). It is conceivable to represent the 2-dimensional solution as a simple expectancy-value model, where the Goal Orientation dimension representing value, with high value represented by Intrinsic Goal Orientation. However, we have avoided the potential conflation of value and goal orientation with the limited data available to explore this relationship directly. Furthermore, the MSLQ was not explicitly designed for EVT, and stretching conclusions to fit this model seem unwarranted at this point.

### Curvilinear Relationships and Test Anxiety

Our primary goal in this study was to expand the literature by exploring the potential for better identifying the relationships among test anxiety and motivational and self-regulated learning constructs. Eysenck et al. (2007) provided a clear call for correcting a long-standing failure of cognitive models for test anxiety to account for conditions in which learners with high levels of test anxiety perform at high levels. Similarly, researchers examining moderation effects among cognitive and emotional constructs in academic settings (e.g., Stowell et al., 2008; Owens et al., 2012) have demonstrated that studies limiting their analyses to simple effects often overlook variations due to moderating effects or variations across contexts.

While the data provide clear evidence of curvilinear effects, it’s important to clarify that traditional methods employed with this data set would have generated significant values and meaningful results. Simple linear effects were significant, but inferior overall in the reported outcomes. Striking a regression line for the “best fit” on a linear scale would indeed generate a line, but the nuance missed that is seen with curvilinear analyses would have masked the true relationships. We anticipate that examination of curvilinear effects in future studies on test anxiety (and related measures) will provide greater precision in isolating the debilitating and facilitative effects of emotional constructs within the academic arenas.

#### Goal Orientation

The significant curvilinear relationship between Goal Orientation and Test Anxiety indicated heightened levels of test anxiety for students who tended to report extreme levels of extrinsic or intrinsic goal orientations. When there were more moderate levels of GO, which indicates a mixed representation for extrinsic and intrinsic goals – as well as a high “task value” orientation in the MDSM – cognitive test anxiety levels were lowest. Compared to recent work with goal orientation that generally indicates that performance goals tend to be overly maladaptive (e.g., Brandmo et al., 2019), these findings demonstrate that a moderate mixture of performance and mastery goals may be more adaptive with respect to test anxiety.

#### Expectancy for Success

The significant “inverted U” pattern observed between Expectancy for Success and Cognitive Test Anxiety was reminiscent of the classic Yerkes-Dodson curve (1908). In this case, the curvilinear relationship exposes an intimate relationship between cognitive test anxiety and uncertainty. When learners are able to reliably predict their performance outcomes, the degree of anxiety was suppressed. Overall, this finding is the most significant theoretical contribution to the test anxiety literature, lending confirmatory empirical evidence to multiple theoretical accounts for test anxiety simultaneously by examining the data in this non-linear approach. In addition, it provides an advancement in the explanation of how these various representations may be fostered and lead to sub-optimal performances.

The value of this curvilinear analysis is the isolation on the key issue in the expectancy–test anxiety relationship. Traditional representations examining linear models conclude that high self-efficacy is associated with lower test anxiety. However, the data in this model suggests that relationship may have been misleading. While it is true that higher self-efficacy tends to be related to higher performance – and lower test anxiety – the data in this case suggest that when it comes to test anxiety, low levels of self-efficacy would not necessarily produce high degrees of anxiety. What has been most clear in these analyses is that the critical issue that tends to drive anxiety upward most readily is a sense of uncertainty or low confidence levels in predicting outcomes. In this way, we see a connection with the findings demonstrating that low levels of perceived control or predicted outcomes for forthcoming evaluative events are likely to be associated with increased test anxiety (e.g., Putwain and Aveyard, 2018; Putwain and Prescod, 2018).

#### Personal Responsibility

A weak statistically significant effect was identified for the Personal Responsibility dimension, which identifies the degree to which learning behaviors or study skills require independent personal engagement. Cognitive test anxiety increased, then plateaued after reaching a critical level of personal responsibility, indicating that those learners who infrequently endorse engaging in individually driven learning activities or frequently employed externalized learning support activities had the lowest levels of cognitive test anxiety. This finding may demonstrate that socially dependent learning strategies provide an “escape” function that allows release from anxiety, consistent with Stowell et al. (2008) findings.

The plateau effect observed with test anxiety once level of personal responsibility reached a critical level may better explain previous findings related to test anxiety and study strategies. There has been ample evidence that students with high-test anxiety engage in various study support strategies, but the divergence when compared to low-test anxious learners is in the quality of chosen strategies (Cassady, 2004; Putwain et al., 2016). Students with high levels of test anxiety are known to employ less efficient processing when engaged in cognitive tasks (Eysenck et al., 2007), primarily presumed to be due to an interference with optimal cognitive functioning (Eysenck and Calvo, 1992; Mattarella-Micke et al., 2011). In contexts where time is not pressed (and sufficient cognitive resources can be tapped), it is possible for learners with academic anxieties to overcome these limitations by extending time on task, resulting in satisfactory performance (Cassady and Johnson, 2002; Owens et al., 2012; Wong et al., 2013). However, in most academic settings, time is a factor and inefficiency will impair overall performance. Furthermore, this extended time of engaging in study occurs within a context of heightened anxiety, promoting a cumulative negative effect for overall affect, increasing the risk for experiencing maladaptive outcomes such as depression and anxiety (Cassady et al., 2019).

#### Active Learning Engagement

There was no curvilinear pattern observed between cognitive test anxiety and the Active Learning dimensional variable. Rather, the data demonstrated a clear and strong inverse linear relationship, demonstrating that as learners were more likely to endorse active learning behaviors, their degree of test anxiety declined. This pattern is once again consistent with research identifying students with high levels of test anxiety are more likely to engage in passive or avoidant coping strategies (e.g., procrastination; Kalechstein et al., 1989; Zeidner and Matthews, 2005), as well as employing more surface-level strategies and engage in repetitive processing that tend to be less effective in promoting deep understanding and learning (Cassady, 2004). Intervention efforts in this domain are promising, provided positive study strategies (e.g., Dunlosky et al., 2013) can be adopted. Several studies have demonstrated that explicit training efforts with test anxious learners can be effective provided the strategies are within their cognitive skill set, the learners recognize the potential for the coping strategy to be successful, and the learners are able to simultaneously manage the other components of test anxiety during the study activities (Lowe et al., 2008; Mowbray, 2012).

## Conclusion

The results of this study continue to refine our understanding of the potential to interrupt the deleterious effects of test anxiety on learners’ experiences in academic domains. Specifically, within a framework consistent with the EIP (Cassady and Thomas, 2020), we propose that the results show promise for future work in both identifying and treating academic anxieties with a more fine-grained approach. Classic studies on effective interventions for those with test anxiety often resulted in weak or moderate effects (e.g., Hembree, 1988; von der Embse et al., 2018). However, we believe this was often due to an over-simplified approach to intervention, where a single intervention was applied to all learners who presented with elevated test anxiety. This approach overlooked the variety of test anxiety profiles (e.g., Zeidner and Matthews, 2005), precluding an individual-specific intervention strategy that would likely have greater utility in practical settings.

Using a multiple domain approach to examining challenges faced by specific learners with test anxiety provides greater promise – identifying interventions that will directly support the learner in the domain(s) where they struggle. That is, we advocate for examining learners’ perceptions of the events (degree of perceived threat and perceived competence to succeed), reviewing the goal structures that have been adopted for the learning event, and identifying specific coping strategies that support optimal performance in meeting those situation-specific goals. While our results demonstrate trend data suggesting that anxiety will peak in situations where the outcome is uncertain, passive learning strategies are adopted, or polarized goal orientations are adopted, truly effective interventions for individual learners will only be achieved when unique profiles are examined and addressed (see de la Fuente et al., 2019, for related perspectives). We see great promise for individual successes in mitigating negative effects associated with test anxiety when these greater levels of refined attention to unique patterns of motivation and self-regulation strategies are used to specify direct interventions.

While the results of this study clearly supported our initial hypotheses that examining relationships among motivational and self-regulated learning dimensions measured by the MSLQ would be better achieved with attention to curvilinear relationships, there are limitations to the current study that require attention in future studies. First, the conditions of our data collection method in this study precluded specific demographic information from individual participants. We know that future studies will add to this literature with the ability to examine differential patterns based on gender, race, prior academic skills, and other variables in the field that have proven instructive. Second, the current study resulted in a dynamic solution for the dimensional scaling of the MSLQ. Repeated examinations with the MSLQ have revealed several competing explanations for representing the structure of the underlying constructs, and our study adds to that list. However, the dimensional analysis results would need to be re-run in future studies to estimate those representations of the dimensions in the MSLQ, as there is no simple translation generated in this procedure. Continued attention to a dimensional representation of the MSLQ is recommended to provide an alternative solution for representing motivation and self-regulated learning in students. Finally, the population from which this sample was drawn was a predominantly female, Caucasian, and from the Midwestern United States. Continued examination of these constructs in more diverse samples would be important for greater generalization.

## Data Availability Statement

The datasets generated for this study will not be made publicly available due to IRB restrictions. Requests to access the datasets should be directed to the corresponding author.

## Ethics Statement

The studies involving human participants were reviewed and approved by the Ball State University Institutional Review Board. The ethics committee waived the requirement of written informed consent for participation.

## Author Contributions

JC conceptualized the data collection process, collected the data, and initiated the interpretation of the findings related to the field. WF conceptualized the analytic approach, conducted all analyses, and collaborated on the interpretation of the findings. JC and WF agreed to the order of authorship but also acknowledge the work was a true collaboration and equitable worth of both authors’ contributions is asserted.

## Conflict of Interest

The authors declare that the research was conducted in the absence of any commercial or financial relationships that could be construed as a potential conflict of interest.

## References

[B1] Armstrong D. A. II Bakker R. Carroll R. Hare C. Poole K. T. Rosenthal H. (2014). *Analyzing Spatial Models of Choice and Judgment with R.* Boca Raton, FL: CRC Press, Taylor & Francis Group.

[B2] Bandura A. (1977). Self-efficacy: toward a unifying theory of behavioral change. *Psychol. Rev.* 84:191. 10.1037/0033-295x.84.2.191 847061

[B3] Bandura A. Schunk D. H. (1981). Cultivating competence, self-efficacy, and intrinsic interest through proximal self-motivation. *J. Pers. Soc. Psychol.* 41 586–598. 10.1037/0022-3514.41.3.586

[B4] Bar-Tal Y. Raviv A. Spitzer A. (1999). The need and ability to achieve cognitive structuring: individual differences that moderate the effect of stress on information processing. *J. Pers. Soc. Psychol.* 77 33–51. 10.1037/0022-3514.77.1.33 10434408

[B5] Bjork R. A. Dunlosky J. Kornell N. (2013). Self-regulated learning: beliefs, techniques, and illusions. *Ann. Rev. Psychol.* 64 417–444. 10.1146/annurev-psych-113011-143823 23020639

[B6] Brady S. T. Hard B. M. Gross J. J. (2018). Reappraising test anxiety increases academic performance of first-year college students. *J. Educ. Psychol.* 110 395–406. 10.1037/edu0000219

[B7] Brandmo C. Bråten I. Schewe O. (2019). Social and personal predictors of test anxiety among Norwegian secondary and postsecondary students. *Soc. Psychol. Educ.* 22 43–61. 10.1007/s11218-018-9461-y

[B8] Brdar I. Rijavec M. Loncaric D. (2006). Goal orientations, coping with school failure and school achievement. *Eur. J. Psychol. Educ.* 21 53–70. 10.1007/BF03173569

[B9] Cassady J. C. (2001). The stability of undergraduate students’ cognitive test anxiety levels. *Pract. Assess. Res. Eval.* 7:20.

[B10] Cassady J. C. (2004). The influence of cognitive test anxiety across the learning-testing cycle. *Learn. Instr.* 14 569–592. 10.1016/j.learninstruc.2004.09.002

[B11] Cassady J. C. (2010). “Test anxiety: contemporary theories and implications for learning,” in *Anxiety in Schools: The Causes, Consequences, and Solutions for Academic Anxieties*, ed. Cassady J. C. (New York, NY: Peter Lang), 5–26.

[B12] Cassady J. C. Boseck J. J. (2008). “Educational psychology and emotional intelligence: toward a functional model for emotional information processing in schools,” in *Emotional Intelligence: Perspectives of Educational and Positive Psychology*, eds Cassady J. C. Eissa M. A. (New York, NY: Peter Lang Publishing), 3–24.

[B13] Cassady J. C. Finch W. H. (2014). Confirming the factor structure of the Cognitive Test Anxiety Scale: comparing the utility of three solutions. *Educ. Assess. J.* 19 229–242. 10.1080/10627197.2014.934604

[B14] Cassady J. C. Finch W. H. (2015). Using factor mixture modeling to identify dimensions of cognitive test anxiety. *Learn. Individ. Differ.* 41 14–20. 10.1016/j.lindif.2015.06.002

[B15] Cassady J. C. Johnson R. E. (2002). Cognitive test anxiety and academic procrastination. *Contemp. Educ. Psychol.* 27 270–295.

[B16] Cassady J. C. Pierson E. E. Starling J. M. (2019). Predicting student depression with measures of general and academic anxieties. *Front. Educ.* 4:11 10.3389/feduc.2019.00011

[B17] Cassady J. C. Thomas C. L. (2020). “Academic anxieties and emotional information processing: identification and interventions to support students with affective disorders,” in *Handbook of Educational Psychology and Students with Special Needs*, eds Martin A. J. Sperling R. A. Newton K. J. (New York, NY: Routledge).

[B18] Cheslock J. J. Kroc R. (2012). “Managing college enrollments,” in *The Handbook for Institutional Researchers*, eds Howard R. Knight B. McLaughlin G. (San Francisco, CA: Jossey-Bass), 221–236.

[B19] Cho M. H. Summers J. J. (2012). Factor validity of the motivated strategies for learning questionnaire (MSLQ) in asynchronous online learning environments (AOLE). *J. Interact. Learn. Res.* 23 5–28.

[B20] de la Fuente J. Martínez-Vicente J. M. Peralta-Sánchez F. J. Garzón-Umerenkova A. Vera M. M. Paoloni P. (2019). Applying the SRL vs. ERL Theory to the Knowledge of Achievement Emotions in Undergraduate University Students. *Front. Psychol.* 10:2070. 10.3389/fpsyg.2019.02070 31620044 PMC6760021

[B21] Deci E. L. Ryan R. M. (2012). “Self-determination theory,” in *Handbook of Theories of Social Psychology*, eds Van Lange P. A. M. Kruglanski A. W. Higgins E. T. (Thousand Oaks, CA: Sage Publications Ltd), 416–436. 10.4135/9781446249215.n21

[B22] Duckworth A. L. Seligman M. E. P. (2005). Self-discipline outdoes IQ in predicting academic performance of adolescents. *Psychol. Sci.* 16 939–944. 10.1111/j.1467-9280.2005.01641.x 16313657

[B23] Dunlosky J. Rawson K. A. Marsh E. J. Nathan M. J. Willingham D. T. (2013). Improving students’ learning with effective learning techniques: promising directions from cognitive and educational psychology. *Psychol. Sci. Public Interest* 14 4–58. 10.1177/1529100612453266 26173288

[B24] Durik A. M. Shechter O. G. Noh M. Rozek C. S. Harackiewicz J. M. (2015). What if I can’t? Success expectancies moderate the effects of utility value information on situational interest and performance. *Motiv. Emot.* 39 104–118. 10.1007/s11031-014-9419-0

[B25] Eysenck M. W. Calvo M. G. (1992). Anxiety and performance: the processing efficiency theory. *Cogn. Emot.* 6 409–434. 10.1080/02699939208409696

[B26] Eysenck M. W. Derakshan N. Santos R. Calvo M. G. (2007). Anxiety and cognitive performance: attentional control theory. *Emotion* 7 336–353. 10.1037/1528-3542.7.2.336 17516812

[B27] Gorges J. Göke T. (2015). How do I know what I can do? Anticipating expectancy of success regarding novel academic tasks. *Br. J. Educ. Psychol.* 85 75–90. 10.1111/bjep.12064 25557305

[B28] Gross J. J. (1998). The emerging field of emotion regulation: an integrative review. *Rev. Gen. Psychol.* 2 271–299. 10.1037/1089-2680.2.3.271

[B29] Gross J. J. (2015). Emotion regulation: current status and future prospects. *Psychol. Inq.* 26 1–15.

[B30] Hastie T. J. Tibshirani R. J. (1990). *Generalized Additive Models.* Orange: Chapman & Hall/CRC.

[B31] Heller M. L. Cassady J. C. (2017). The impact of perceived barriers, academic anxiety and resource management strategies on achievement in first year community college students. *J. First Year Exp. Stud. Trans.* 29 9–32.

[B32] Hembree R. (1988). Correlates, causes, effects and treatment of test anxiety. *Rev. Educ. Res.* 58 47–77. 10.3102/00346543058001047

[B33] Kalechstein P. Hocevar D. Zimmer J. W. Kalechstein M. (1989). “Procrastination over test preparation and test anxiety,” in *Advances in Test Anxiety Research*, Vol. 6 eds Schwarzer R. van der Ploeg H. M. Spiel- berger G. D. (Lisse: 63–76).

[B34] Kessler R. C. Chiu W. T. Demler O. Merikangas K. R. Walters E. E. (2005). Prevalence, severity, and comorbidity of 12-month DSM-IV disorders in the National Comorbidity Survey Replication. *Arch. Gen. Psychiatry* 62 617–627. 10.1001/archpsyc.62.6.617 15939839 PMC2847357

[B35] Kitsantas A. Zimmerman B. J. (2009). College students’ homework and academic achievement: the mediating role of self-regulatory beliefs. *Metacogn. Learn.* 4 97–110. 10.1007/s11409-008-9028-y

[B36] Lazarus R. Folkman S. (1987). Transactional theory and research on emotions and coping. *Eur. J. Pers.* 1 141–169. 10.1002/per.2410010304

[B37] Liebert R. M. Morris L. W. (1967). Cognitive and emotional components of test anxiety: a distinction and some initial data. *Psychol. Rep.* 20 975–978. 10.1108/eb026408 6042522

[B38] Lowe P. Lee S. Witteburg K. Prichard K. Luhr M. Cullinan C. (2008). The test anxiety inventory for children and adolescents (TAICA): evaluation of the psychometric properties of a new multidimentional measure of test anxiety among elementary and secondary school students. *J. Psychometr. Assess.* 26 215–230. 10.1177/0734282907303760

[B39] Mattarella-Micke A. Mateo J. Kozak M. N. Foster K. Beilock S. L. (2011). Choke or thrive? The relation between salivary cortisol and math performance depends on individual differences in working memory and math anxiety. *Emotion* 11 1000–1005. 10.1037/a0023224 21707166

[B40] Mayer R. E. (2014). “Cognitive theory of multimedia learning,” in *Cambridge Handbooks in Psychology. The Cambridge Handbook of Multimedia Learning*, ed. Mayer R. E. (Cambridge: Cambridge University Press), 43–71. 10.1017/CBO9781139547369.005

[B41] Mowbray T. (2012). Working memory, test anxiety and effective interventions: a review. *Aust. Educ. Dev. Psychol.* 29 141–156. 10.1017/edp.2012.16

[B42] Naveh-Benjamin M. McKeachie W. J. Lin Y. (1987). Two types of test-anxious students: support for an information processing model. *J. Educ. Psychol.* 79 131–136. 10.1037/0022-0663.79.2.131

[B43] O’Carroll P. J. Fisher P. (2013). Metacognitions, worry and attentional control in predicting OSCE performance test anxiety. *Med. Educ.* 47 562–568. 10.1111/medu.12125 23662873

[B44] Owens M. Stevenson J. Hadwin J. A. Norgate R. (2012). When does anxiety help or hinder cognitive test performance? The role of working memory capacity. *Br. J. Psychol.* 105 92–101. 10.1111/bjop.12009 24387098

[B45] Pekrun R. (2006). The control-value theory of achievement emotions: assumptions, corollaries, and implications for educational research and practice. *Educ. Psychol. Rev.* 18 315–341. 10.1007/s10648-006-9029-9

[B46] Pintrich P. R. (2004). A conceptual framework for assessing motivation and self-regulated learning in college students. *Educ. Psychol. Rev.* 16 385–407. 10.1007/s10648-004-0006-x

[B47] Pintrich P. R. Smith D. A. F. Garcia T. G. McKeachie W. J. (1991). *A Manual for the Use of the Motivated Strategies for Learning Questionnaire (MSLQ).* Ann Arbor, MI: National Center for Research to Improve Postsecondary Teaching.

[B48] Putwain D. W. (2007). Test anxiety in UK schoolchildren: prevalence and demographic patterns. *Br. J. Educ. Psychol.* 77 579–593. 10.1348/000709906X161704 17908376

[B49] Putwain D. W. (2019). An examination of the self-referent executive processing model of test anxiety: control, emotional regulation, self-handicapping, and examination performance. *Eur. J. Psychol. Educ.* 34 341–358. 10.1007/s10212-018-0383-z

[B50] Putwain D. W. Aveyard B. (2018). Is perceived control a critical factor in understanding the negative relationship between cognitive test anxiety and examination performance? *Sch. Psychol. Q.* 33 65–74. 10.1037/spq0000183 27831699

[B51] Putwain D. W. Daly T. Chamberlain S. Saddredini S. (2016). “Sink or swim”: buoyancy and coping in the test anxiety and academic performance relationship. *Educ. Psychol.* 36 1807–1825. 10.1080/01443410.2015.1066493

[B52] Putwain D. W. Prescod M. (2018). Is reducing uncertain control the key to successful test anxiety for Secondary school students? Findings from a randomized control trial. *Sch. Psychol. Q.* 33 283–292. 10.1037/spq0000228 29094957

[B53] R Core Team (2019). *R: A Language and Environment for Statistical Computing.* Vienna: R Foundation for Statistical Computing.

[B54] Raffety B. D. Smith R. E. Ptacek J. T. (1997). Facilitating and debilitating trait anxiety, situational anxiety, and coping with an anticipated stressor: a process analysis. *J. Pers. Soc. Psychol.* 72 892–906. 10.1037/0022-3514.72.4.892 9108702

[B55] Rojas J. S. Furlan L. A. (2017). Achievement emotions and achievement goals in support of the convergent, divergent, and criterion validity of the Spanish-Cognitive Test Anxiety Scale. *Int. J. Educ. Psychol.* 6 67–92.

[B56] Sarason I. G. (1986). “Test anxiety, worry, and cognitive interference,” in *Self-Related Cognitions in Anxiety and Motivation*, ed. Schwarzer R. (Hillsdale, NJ: Lawrence Erlbaum), 19–34.

[B57] Schunk D. H. (1990). Goal setting and self-efficacy during self-regulated learning. *Educ. Psychol.* 26 207–231.

[B58] Schunk D. H. Zimmerman B. J. (2003). “Self-regulation and learning,” in *Handbook of Psychology*, Vol. 7 eds Reynolds W. M. Miller G. E. (Hoboken, NJ: Wiley), 59–78.

[B59] Shim S. S. Ryan A. M. Cassady J. C. (2011). Self-esteem change during the first year of college: the role of achievement goals. *Educ. Psychol.* 32 149–167. 10.1080/01443410.2011.627837

[B60] Stowell J. R. Tumminaro T. Attarwala M. (2008). Moderating effects of coping on the relationship between test anxiety and negative mood. *Stress Health* 24 313–324. 10.1002/smi.1186

[B61] Thomas C. L. Cassady J. C. Finch W. H. (2017a). Identifying severity standards on the cognitive test anxiety scale using Latent class and cluster analysis. *J. Psychoeduc. Assess.* 36 492–508. 10.1177/0734282916686004

[B62] Thomas C. L. Cassady J. C. Heller M. L. (2017b). The influence of emotional intelligence, cognitive test anxiety, and coping strategies on undergraduate academic performance. *Learn. Individ. Differ.* 55 40–48. 10.1016/j.lindif.2017.03.001

[B63] von der Embse N. P. Jester D. Roy D. Post J. (2018). Test anxiety effects, predictors, and correlates: a 30-year meta-analytic review. *J. Affect. Disord.* 227 483–493. 10.1016/j.jad.2017.11.048 29156362

[B64] Weiner B. (2018). The legacy of an attribution approach to motivation and emotion: a no-crisis zone. *Motiv. Sci.* 4 4–14. 10.1037/mot0000082

[B65] Wells A. Matthews G. (1996). Modelling cognition in emotional disorder: The S-REF model. *Behav. Res. Ther.* 34 881–888. 10.1016/s0005-7967(96)00050-2 8990539

[B66] Wigfield A. Eccles J. S. (2002). “The development of competence beliefs, expectancies for success, and achievement values from childhood through adolescence,” in *A Volume in the Educational Psychology Series. Development of Achievement Motivation*, eds Wigfield A. Eccles J. S. (Cambridge, MA: Academic Press), 91–120. 10.1016/B978-012750053-9/50006-1

[B67] Wong I. Mahar D. Titchener K. Freeman J. (2013). The impact of anxiety on processing efficiency: implications for the Attentional Control Theory. *Open Behav. Sci. J.* 7 7–15. 10.2174/1874230001307010007

[B68] Yerkes R. M. Dodson J. D. (1908). The relation of strength of stimulus to rapidity of habit-formation. *J. Comp. Neurol. Psychol.* 18 459–482. 10.1002/cne.920180503

[B69] Zeidner M. Matthews G. (2005). “Evaluation anxiety,” in *Handbook of Competence and Motivation*, eds Elliot A. J. Dweck C. S. (London: Guildford Press), 141–163.

[B70] Zeidner M. Saklofske D. (1996). “Adaptive and maladaptive coping,” in *Handbook of Coping: Theory, Research, Applications*, eds Zeidner M. Endler N. S. (Hoboken, NJ: John Wiley & Sons), 505–531.

[B71] Zimmerman B. J. (2002). Becoming a self-regulated learner: an overview. *Theor. Pract.* 41 64–72. 10.1207/s15430421tip4102_2

[B72] Zohar D. (1998). An additive model of test anxiety: role of exam-specific expectations. *J. Educ. Psychol.* 90 330–340. 10.1037/0022-0663.90.2.330

